# Searching for 3D structural models from a library of biological shapes using a few 2D experimental images

**DOI:** 10.1186/s12859-018-2358-0

**Published:** 2018-09-12

**Authors:** Sandhya P. Tiwari, Florence Tama, Osamu Miyashita

**Affiliations:** 1 Computational Structural Biology Unit, RIKEN Center for Computational Science, Kobe, Japan; 2 Graduate School of Science, Department of Physics & Institute of Transformative Bio-Molecules (WPI-ITbM), Nagoya University, Nagoya, Japan

**Keywords:** Electron microscopy, Image analysis, Single particle analysis, Biomolecular structure

## Abstract

**Background:**

Advancements in biophysical experimental techniques have pushed the limits in terms of the types of phenomena that can be characterized, the amount of data that can be produced and the resolution at which we can visualize them. Single particle techniques such as Electron Microscopy (EM) and X-ray free electron laser (XFEL) scattering require a large number of 2D images collected to resolve three-dimensional (3D) structures. In this study, we propose a quick strategy to retrieve potential 3D shapes, as low-resolution models, from a few 2D experimental images by searching a library of 2D projection images generated from existing 3D structures.

**Results:**

We developed the protocol to assemble a non-redundant set of 3D shapes for generating the 2D image library, and to retrieve potential match 3D shapes for query images, using EM data as a test. In our strategy, we disregard differences in volume size, giving previously unknown structures and conformations a greater number of 3D biological shapes as possible matches. We tested the strategy using images from three EM models as query images for searches against a library of 22750 2D projection images generated from 250 random EM models. We found that our ability to identify 3D shapes that match the query images depends on how complex the outline of the 2D shapes are and whether they are represented in the search image library.

**Conclusions:**

Through our computational method, we are able to quickly retrieve a 3D shape from a few 2D projection images. Our approach has the potential for exploring other types of 2D single particle structural data such as from XFEL scattering experiments, for providing a tool to interpret low-resolution data that may be insufficient for 3D reconstruction, and for estimating the mixing of states or conformations that could exist in such experimental data.

**Electronic supplementary material:**

The online version of this article (10.1186/s12859-018-2358-0) contains supplementary material, which is available to authorized users.

## Background

Biophysical techniques such as X-ray crystallography, Nuclear Magnetic Resonance and Electron Microscopy (EM) have provided us with the ability to visualize biological cells and molecules in three-dimensions (3D). Single particle EM techniques in particular have paved the way for larger, non-crystallizable complexes to be probed with increasing resolution [1]. X-ray free electron laser (XFEL) scattering is another such novel technique that will create new opportunities to view biological molecules and larger assemblies that have eluded us thus far [2].

However, most experimental methods do not directly provide 3D models. Some provide two-dimensional (2D) images (e.g. EM, XFEL) while others provide even lower dimensional data (e.g. small angle X-ray scattering (SAXS), fluorescence resonance energy transfer). Such experimental data needs to be further analyzed computationally to produce a 3D model. In the reconstruction of 3D models in single particle analysis, the orientation angles of the 2D images of sample have to be determined from the signal in the noisy raw data [1]. Complex computational algorithms are required to analyze large amounts of experimental data of reasonable quality to produce good quality 3D structures from EM [3] and XFEL [1, 4]. Moreover, in X-ray techniques, the phase information is required for structure reconstruction, which is a problem that is still difficult to solve [5–7]. Extensive efforts have been devoted to development of software packages to analyze single-particle EM data [8–13] and also for new XFEL data [14]. Thus, a large number of experimental images with clean samples, homogenous structures and computational processing are required for 3D reconstruction.

However, there are cases where the experimental data are insufficient in quality and quantity for de novo 3D reconstruction. In such cases where the resolution or amount of the data is low, hybrid approaches that combine computational modeling with experimental data are required to obtain the 3D structure models. One application is the modeling from low-resolution cryo-EM maps, where computational modeling tools are used to generate detailed structural models that conform to low-resolution maps. Multiple approaches have been developed and successfully applied to experimental data to model conformational changes [15–27]. The hybrid approach can be also extended to XFEL data [28–30] and also combine multiple experimental data to increase the applicability [31–33].

Another type of approaches uses prebuilt database of possible structures and expected experimental observations. The DARA webserver is a tool that can identify structural neighbors by matching experimental and simulated small-angle X-ray scattering (SAXS) data to a database of pre-computed simulated SAXS profiles of known structures [34]. SASTBX is another tool that uses a database of 3D shapes to propose 3D models matching the SAXS data [35]. However, there are no computational tools available to quickly predict 3D shapes directly from a few XFEL or EM experimental 2D images, which we aim to address in our study.

**Fig. 1:**
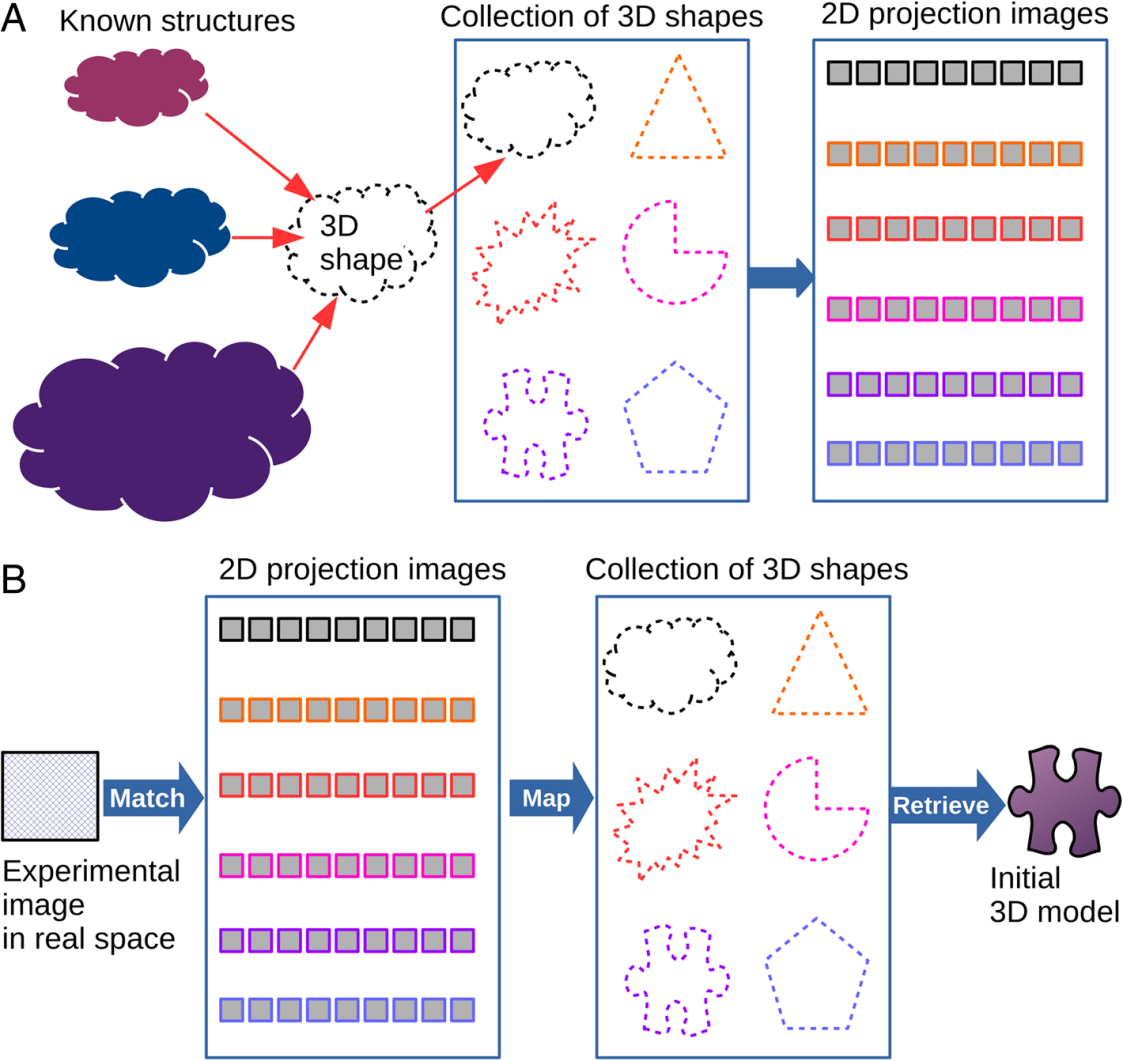
**a** Overview of building the 2D projection image library from 3D biological shapes. Known structures are resized to have the same particle volume and are aligned with each other to determine their similarity. Representative shapes are picked for each shape type and projection images are simulated based on them for the 2D projection image library. **b** Overview of finding candidate 3D models from a few 2D experimental images in real space. The input image is aligned against the library of images, and the close matches are mapped to their corresponding 3D models, resulting in a potential 3D candidate model

We present a hybrid approach to search for 3D models by comparing some experimental image data to the projection images from existing structural data (Fig. 1). Here, an experimental image can be quickly aligned to all the images in the library, and the best matches can be mapped on to their corresponding 3D models, retrieving a possible matching 3D shape (Fig. 1b). As part of the strategy, we construct a set of 3D biological shapes, where we include a novel step of resizing the 3D models to have the same volumetric size, allowing for the possibility for a small novel protein to have a similar shape to a large protein complex (Fig. 1a). In other words, we use existing known structures to create a library of low-resolution shapes by discarding the original molecular compositions. Focusing on shape over volumetric size increases the coverage of the library to a larger number of possible biological shapes. We hypothesize that by disregarding volume, we can obtain the 3D shape of a previously uncharacterized sample from our library of shapes without prior information on its identity or sequence information. We use existing 2D image alignment algorithms for EM data analysis (XMIPP) [8] and a 3D structure fitting program (GMFIT) [36], which aligns coarse-grained 3D representation of structure data from X-ray crystallography and EM. We tested our strategy using single particle models from the Electron Microscopy Database (EMDB), and simulated 2D projection images in real space from EM models that are resized to have the same actual volume.

## Methods

### Preparation of 3D model datasets from EMDB

In this study, we used single particle EM data as our primary source of biological shapes. In order to select optimal parameters for our protocol, we first assembled a small test dataset of 25 EM models from the single particle entries in the Electron Microscopy Pilot Image Archive (EMPIAR) [37]. Using this small dataset, we first developed formalisms to analyze the relation between 3D models and 2D images to select suitable parameters, and then tested our protocol on an expanded dataset of 250 EM single particle models obtained from the Electron Microscopy Data Bank (EMDB) (Additional file 1: Table S1, “Resizing and aligning 3D models” section) [38].

### Resizing and aligning 3D models

In this approach, we aim to provide a tool that proposes low-resolution “shapes”, regardless of the volumetric size, from a limited number of query images. In other words, if two volumes have different sizes but the same shape, they should be considered as a single entry. Thus, we resized the volumes of the EM models where they have the same approximate volume and compared their similarity in shape by aligning them. To align the EM models, we used the program GMFIT [36], which has also been used by the web-tool Omokage Search to visualize the 3D alignment of pairs of structures [39]. GMFIT uses Gaussian Mixture Model (GMM) to extract the overall shapes of the models across different experimental sources, such as X-ray Crystallography or EM. The GMM is a function consisting of several Gaussian distribution functions (GDFs), and the overlap between two GDFs can be obtained analytically, allowing volumes to be aligned quickly.

First, all 3D models from the EMPIAR dataset were initially resized to normalize every EM density map to have the same grid size of 1 Å, and a uniform cubic dimension of 100 Å per side, using the XMIPP image_resize function with spline interpolation (14). For the small dataset, every model was converted to a Gaussian mixture model (GMM) with 20 GDFs and a maximum number of voxels per cubic dimension set to 64, as recommended by GMFIT to speed up the conversion. For the whole EMDB single particle dataset, every model was converted to a Gaussian mixture model (GMM) with 40 GDFs and a maximum number for voxels of each axis was again set to 64. In both alignment procedures, we retrieved the volume information from the GMMs constructed automatically from the initial resized models. The retrieved initial volume was then used to resize each EM model again using the XMIPP image_resize function so that all the models have the same particle volume (50^3^ Å^3^) (Eq. 1). This volume was selected so that sufficient space exists around the molecule, for all types of represented EM maps, in the 2D projection images, which are 64 by 64 pixels.

1 $$ {D}_{new}={D}_{old}\times \kern0.5em \frac{D_{ref}}{\sqrt[3]{V_{old}}} $$

where *D*_new_ is the new axis dimension, *D*_old_ is 100, *V*_*old*_ is the volume of the EM model as retrieved from its GMM and *D*_ref_ is 50 Å.

The newly resized EM models with the approximate particle volume of 50^3^ Å^3^ were again converted to GMMs using the same parameters, and superposed with each other using GMFIT. We used the correlation coefficient (CC) as the 3D structure similarity measure (Eq. 2)

2 $$ CC=\frac{\int_{-\infty}^{\infty }{f}_A(r){f}_B(r) dr}{\sqrt{\int_{-\infty}^{\infty }{f}_A^2(r) dr{\int}_{-\infty}^{\infty }{f}_B^2}(r) dr} $$

where *f*_A_ (***r***) is the distribution function of one GMM (A), and *f*_B_ (***r***) is the distribution function of the other GMM (B) [39]. The CC values range from − 1 to 1, where 1 indicates maximum similarity.

### Generating 2D projection images from resized 3D models

The 2D projection image library was created from 3D EM density maps in XMIPP, using the angular_project_library program [8]. The angular_project_library program takes a 3D volume and calculates 2D projection images from different orientations. The surface of the volume is divided into a triangular grid based on an icosahedron and sampled evenly. In the small dataset, 196 2D projection images were created for each of the 25 EM models in the initial test dataset (4900 images in total). In the expanded set of 250 EM models used to test the match retrieval, 91 projection images were created per model (22750 in total) in order to reduce the computational cost without compromising sufficient coverage of the 3D shape. Every 2D projection image generated is 64 by 64 pixels in size.

To test the developed match retrieval algorithm on the expanded dataset, we chose 3 example models that are not present in the 2D projection image library as test cases: EMD-3347 (T20S proteasome), EMD-2275 (Yeast 80S ribosome) and EMD-2326 (GroEL/ES with ligand). For each of the three models, we chose 5 random images from the stack of images created in the protocol above and used them as input images.

### Aligning and assessing the similarity between 2D projection images

The alignment of the 2D projection images were performed using a modified version of XMIPP’s align2d utility [8] to assess their similarity. We aligned all the images in a stack directly against one of the images as a fixed query reference, where we retrieved the maximum correlation coefficient (CC) for each image against that reference. The maximum CCs retrieved here were then used to calculate the overall match score between the input and each of the EMDB ID represented in the projection image library.

### Statistical analysis

All the statistical analyses were performed using R package version 3.2.2 [40].

#### Clustering

We performed hierarchical clustering to group the EM models based on their 3D structure similarity, and the overlap in their 2D projection images. The 3D and 2D hierarchical clustering were performed using the following eq. (3) as the distance, *d*:

3 $$ d=\sqrt[2]{\left(1-{CC}^2\right)}\kern0.5em $$

where *CC* is the pairwise correlation coefficient from the GMFIT or align2d alignment. The hierarchical clustering was performed using the ward.D clustering algorithm [41].

For the small dataset, in addition to the comparison of the 3D shapes, we sought to assess the similarity between the 25 EMDB IDs based solely on their 2D projection image set (196 images each). We first calculated the submatrix of CCs between images for one EM model against images from all EM models (196 by 4900 CCs), which we used to calculate the Pearson’s correlation coefficient (PCC). Using the PCCs, we then performed hierarchical clustering with the same parameters as stated above to gain insight into the overall similarity between the 2D projection images per EMDB ID.

#### Multidimensional scaling

In order to visualize the similarity between each individual 2D projection image in the small dataset, we performed classical multidimensional scaling analysis (MDS) on the 4900 by 4900 pairwise score matrix constructed with the 2D image alignment CCs (see “Aligning and assessing the similarity between 2D projection images” section; Fig. 4). Classical multidimensional scaling presents the data in *k*-dimensional space, such that the distances between them are approximately equal to their dissimilarities. In the MDS analysis, the parameter *k* was set to 2, such that only 2 dimensions are calculated, after assessing the goodness of fit and the associated eigenvalues. For the visualization, we divide the resulting plot into cells that are 0.1 by 0.1 unit and count the number of images and the average CC in each cell (Fig. 4b). We populate these cells by selecting a representative image with the highest occurring EMDB-ID in that cell (Fig. 5).

### 3D model match retrieval protocol

The purpose of such database is to identify corresponding 3D shapes to a given query image based on its similarity to the images in the 2D projection image library. In order to retrieve matches for a given query 2D projection image, we defined a match score based on the 2D alignment CCs. First, the CCs are normalized as Z-scores, calculated as:

4 $$ Z\left(i,j\right)=\frac{CC_{i,j}-{\mu}_i}{\sigma_i}\kern0.5em $$

where *CC*_*i,j*_ is the align2d correlation coefficient between a given input image *i* and a projection image from the library *j*, μ_i_ is the mean pairwise correlation coefficient for input image *i* and σ_i_ is the standard deviation. Then, for each EMDB ID in the image library, denoted by *n,* the top ten Z-scores are summed per input image *i,* giving *X*_*i*_^*n*^. For *x* number of input images, the sum of top ten Z-scores per EMDB ID, *S*_*n*_ is given by:

5 $$ {S}_n=\sum \limits_{i=1}^x{X}_i^n $$

The final match score *T*_*n*_ for the EMDB ID *n* is given by:

6 $$ {T}_n=\frac{S_n-{\mu}_S}{\sigma_S}\kern0.5em $$

where μ_S_ is the average *S*_*n*_ for all EMDB IDs and, *σ*_*S*_ is the corresponding standard deviation.

## Results

### Analysis of single particle entries in EMDB

The single particle EMDB entries retrieved in August 2016 can be described as having mostly up to 3 unique components per EM map (Additional file 1: Figure S1A). The resolutions of the maps are spread between 1.72 Å and 78.1 Å (Additional file 1: Figure S1B). Most of these entries are for various kinds of proteins, followed by viruses (Additional file 1: Figure S1C). The EMPIAR dataset of 25 models has 20 protein structures, 1 virus, and 4 ribosome structures in different complex states.

### Initial analysis of the 25 EM model test dataset

#### Comparing size normalized 3D shapes

**Fig. 2:**
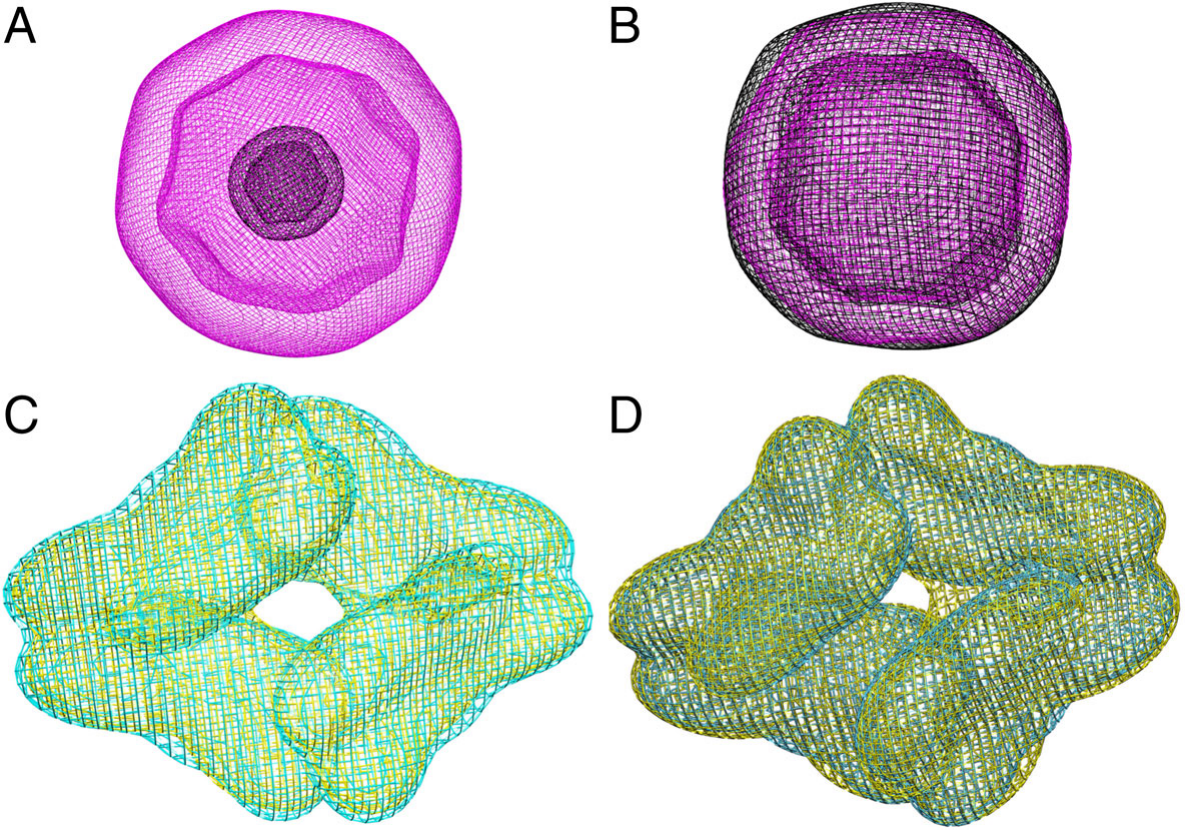
Superposed GMMs of spherical proteins and beta-galactosidases represented as wire surfaces. In (**a**) horse apoferritin protein EMD-2788 is represented in black and Brome mosaic virus EMD-6000 is in magenta and are illustrated to show the large difference in their sizes. In (**b**) EMD-2788 (black) and EMD-6000 (magenta) are resized to have similar particle volumes, as shown by the greater fit of their superposition. **c** shows the pairing of two beta-galactosidases EMD-5995 (yellow) and EMD-2824 (cyan) before they have been resized, where EMD-2824 is slightly larger than EMD-5995, while (**d**) shows the beta-galactosidases pairing after they have been resized to have the same volume

In order to extract the overall 3D biological shape, we resized the EM models to have the same particle volume. The advantage of doing this is two-fold: to decrease redundancy of shapes, and to normalize discrepancies between the models. For example, when we examine the GMMs surfaces, we identified two spherical shapes, Brome mosaic viral capsid (EMD-6000) and horse spleen apoferritin (EMD-2788) (Fig. 2a). When we resized the two spherical shapes, we found that the similarity between the shapes was very high at correlation coefficient of 0.932 (Fig. 2b). In the second example, we assessed the pairing of the two beta-galactosidases EMD-5995 (yellow) and EMD-2824 (cyan), which have slight differences in their overall size (Fig. 2c). When we resized EMD-5995 and EMD-2824, the correlation coefficient improved from 0.971 to 0.982 (Fig. 2d).

**Fig. 3:**
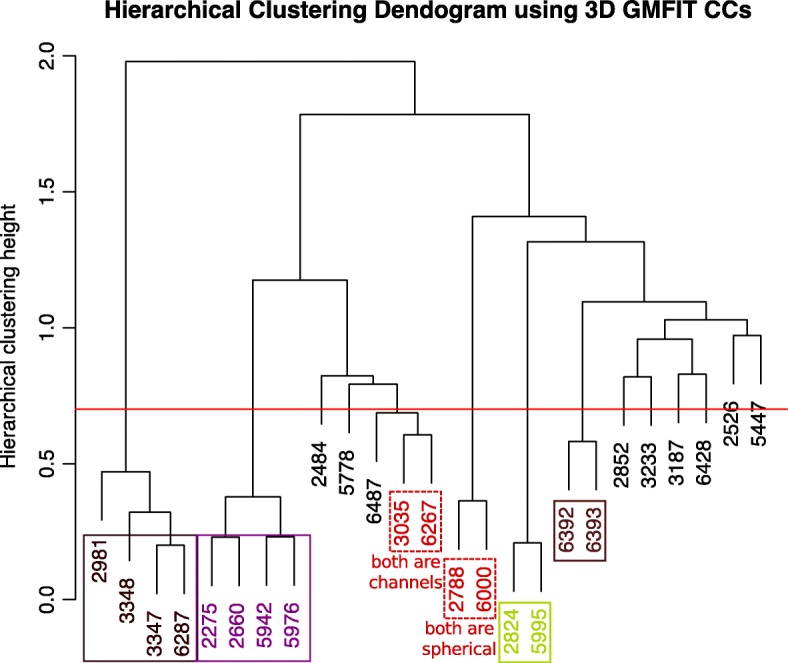
Hierarchical clustering dendrogram of 3D similarity (GMFIT correlation coefficients) between 25 entries in the small test dataset. EMD-2788 and EMD-6000, which are both spherical shapes group together (red dashed box), as well as the EMD-3035 and EMD-6267 which are unrelated membrane channels (orange dashed box), the beta-galactosidases (EMD-5995 and EMD-2824; green solid box), the proteasomes (EMD-2981, EMD-3348, EMD-3347, EMD-6287; purple solid box, the ribosomes (EMD-2275, EMD-2660, EMD-5942 and EMD-5976; magenta solid box) and the tubulin cofactor complexes (EMD-6392, EMD-6393; brown box). We observe that all these related structures fall below the clustering height of 0.7 (red line)

In the hierarchical clustering of the 3D similarity scores, we noted that the same type of structures, EMD-5995 and EMD-2824 (beta galactosidases), EMD-6392 and EMD-6393 (tubulin co-factor complexes), EMD-2275, EMD-2660, EMD-5942 and EMD-5976 (ribosomes) and EMD-2981, EMD-3348, EMD-3347, EMD-6287 (proteasomes), cluster together at lower heights below 0.7 on the dendrogram (Eq. 3 and Fig. 3), indicating a higher level of similarity within those groups. Moreover, we find that EMD-2788 and EMD-6000, which are both spherical shapes as mentioned above, cluster together (Fig. 3; red dashed box), albeit at a greater height than other cluster groups that consist of the same type of proteins. This shows that the overall 3D shape can be sufficiently described by GMMs, and that similar 3D biological shapes can cluster together regardless of structure type.

#### 2D projection image comparisons

**Fig. 4:**
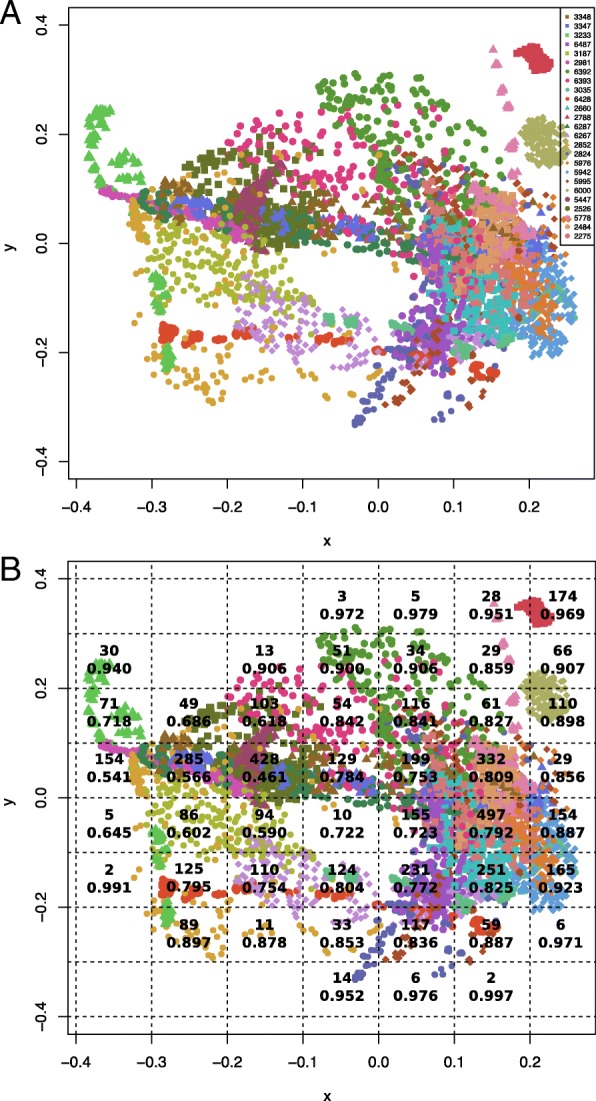
**a** Multidimensional scaling plot of the small dataset 2D projection images, as calculated from their pairwise similarity scores (maximum correlation coefficients) from XMIPP align2D. Each point represents a 2D projection image in the dataset, colored according to its EMDB ID. **b** Number of multidimensional scaling points from the small dataset 2D projection images in each 0.1 by 0.1 unit cell (above) and mean pairwise correlation coefficients between the images in each cell (below)

We performed multi-dimensional scaling (MDS) to visualize the (dis)similarities between the 2D projection images, based on their alignment CCs, in two dimensions. While most small dataset EMDB IDs have their corresponding 2D projection images grouped closely together, some models such as EMD-2852, EMD-5995 and EMD-6393 have their 2D projection images spanning a large region of the two-dimensional MDS space (Fig. 4a). EMD-2852 is a mitochondrial F-type ATP synthase dimer with a flat crown-like shape, EMD-5995 is a beta-galactosidase (Fig. 2c, yellow) that has a quasi-rhombohedral shape with several different faces, while EMD-6393 is a low-resolution EM model (24 Å) of a tubulin-cofactor complex made up of 5 components resulting in a highly irregular shape. The observable differences in the outline of these shapes from different angles could explain the diversity in their corresponding 2D projection image sets.

**Fig. 5:**
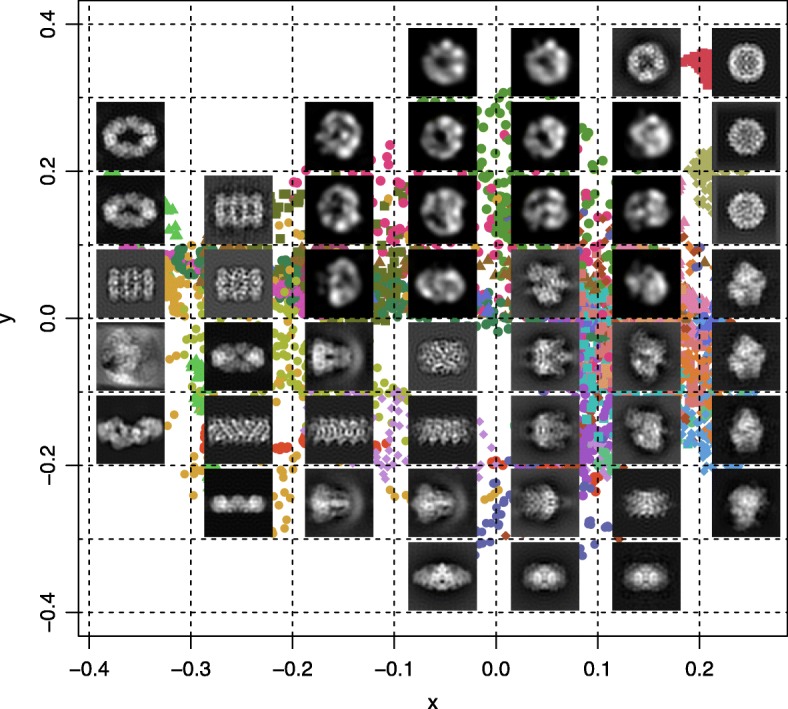
Representative 2D projection images on multidimensional scaling plot. For each 0.1 by 0.1 unit cell in the multidimensional scaling plot of the small dataset (Fig. 4), a representative 2D projection image is selected from the EMDB ID with the largest number of images occurring in each cell and overlaid

To analyze the overlap between the points, we arbitrarily divided the MDS plot into 0.1 by 0.1 unit cells. We counted the number of points within each cell as well as the mean of the 2D similarity scores (CCs) between the points in each cell (Fig. 4b). To observe the distribution of the 2D projection image types on the MDS coordinate space, we display a representative image from each cell (Fig. 5). We find that the shape types on the MDS plot goes from linearly-shaped (Fig. 5; bottom left-hand corner) to circular-shaped (Fig. 5; top right-hand corner). Moreover, the most populated cells consist of irregular globular shapes corresponding to the type obtained from ribosomes, while the flat cylinder shapes are also abundant in the data (Additional file 1: Figure S2). This result indicates that due to the large overlap between many of the 2D projection, it is difficult to distinguish between 3D models based on a single 2D image. Thus, we would require a combination of 2D projection images to increase the possibility of capturing the overall 2D image profile belonging to particular 3D shape.

**Fig. 6:**
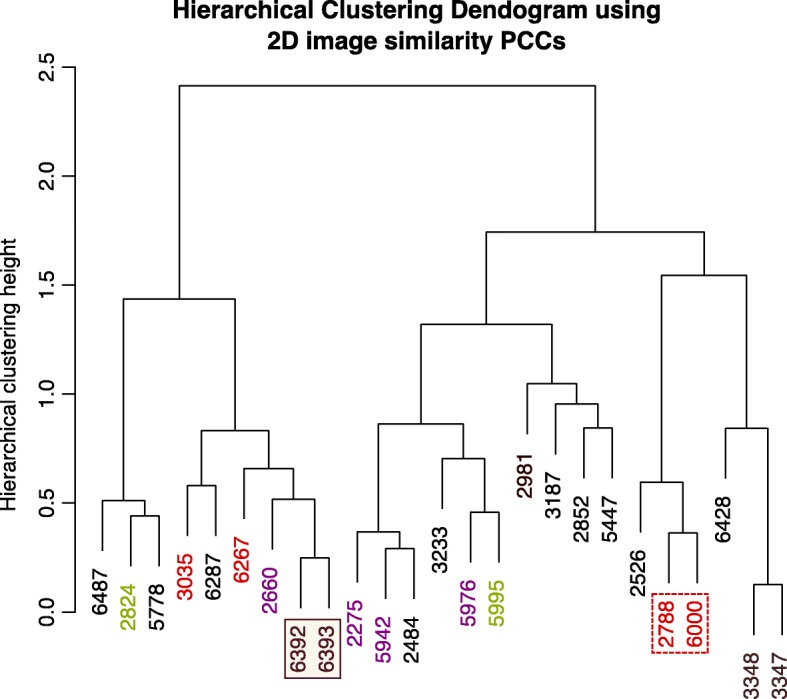
Hierarchical cluster dendrogram of 2D image alignment similarity scores, converted to Pearson’s Correlation Coefficients (PCCs), from 25 small dataset EM models. The color-coding of the individual EMDB IDs follows Fig. 3, while the boxes indicate that the tubulin cofactor complexes (EMD-6392, EMD-6393; brown box), and the spherical shapes (EMD-2788 and EMD-6000; red dashed box) group together as previously observed

In addition, we determined how the EM models cluster with each other based on the information provided by their 2D projection image similarity. By clustering the Pearson’s correlation coefficient calculated using 2D alignment CCs, we compared the overall relationship between 196 images per EMDB model to all other 2D projection images in the small dataset. In general, there is reasonable agreement between the 3D and the 2D hierarchical clustering results, and this agreement is largely dependent on the complexity of the overall 3D shape. When we compare the hierarchical clustering from the 3D analysis (Fig. 3) to the 2D analysis (Fig. 6) in detail, we find that there is agreement between closely related pairs of models such as EMD-3348 and EMD-3347, EMD-6392 and EMD-6393, and spherical shapes like EMD-2788 and EMD-6000. However, we observe that the clustering between EMD-6287 with EMD-3348 and EMD-3347 (proteasomes), EMD-2275 and EMD-2660 (ribosomes), EMD-2824 and EMD-5995 (beta-galactosidases) seen in the 3D alignment is lost in the 2D analysis. The differences in the features between the structures of these complex shapes are captured in the projection images, which probably emphasize the differences between the projection image sets when they are aligned. When we performed the same analysis with a varying the number of 2D projections images per EM model (58, 101, 203 and 406), we observed that a lower number about 100 projection images per model is sufficient to differentiate between them (Additional file 1: Figure S4). Thus in the following analysis with a larger dataset, 91 projection images per EM model were used.

### Retrieving matches for a query from a representative library of 2D images

Here, we constructed a large 3D model dataset to build a library of 2D projection images to test our match retrieval protocol. Protein, virus, and ribosomal structure types make up the bulk of all single particle EMDB entries (Additional file 1: Figure S3A). After performing hierarchical clustering on the GMFIT CCs obtained from the 3D alignment using GMMs with 40 Gaussian distribution points, we reduced the number of EMDB single particle models from 3144 to 1572, by setting the median height of 0.574 as the cut-off. The median height, which is the point at which half of the structures cluster in the hierarchical clustering, was chosen as a reasonable point to remove structures with high similarity. When we examined a few of the groups that form below the cut-off of 0.574, we find that they share 3D GMFIT CC of 0.9 and above. The representation of the molecular types remains largely the same in terms of percentage in the reduced dataset, except for a slight increase in the percentage of protein structure types, and reduction in ribosomal structures for both prokaryotes and eukaryotes (Additional file 1: Figure S3B). To build the 2D projection library, we expanded the test dataset from 25 to 250 EM models; 238 EM models from the reduced EMDB dataset were randomly selected and then added to the 12 reduced EM models from the 25 EMPIAR-EMDB entries. We find that the proportion of structure types does not change significantly by selecting 250 EM models and is representative of the type of shapes in the whole EMDB (Additional file 1: Figure S3C).

#### 2D image comparison – Searching for matches in three different test-cases

**Fig. 7:**
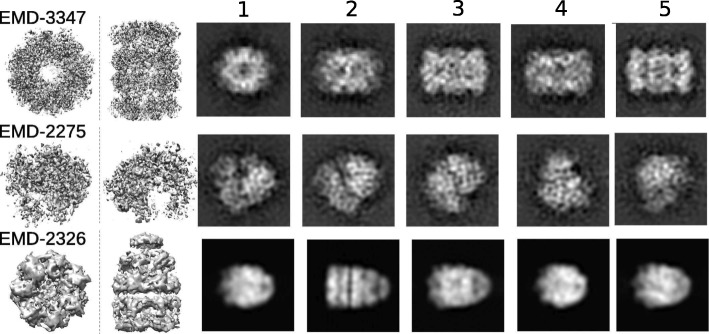
Five random 2D projection images used as input for testing 3D candidate model search from EMD-3347, EMD-2275 and EMD-2326. Two views of each EM model are displayed below the model name (left) and the input projection images numbered 1 to 5 are displayed in the same row (right)

Based on the analysis we performed on the small dataset (“Background” section), we showed that a) 2D projection images from a given 3D model can be diverse and b) there is a large overlap between most of the projection images. These results indicate that using a single input image to retrieve a 3D model is expected to be unreliable. Thus, we used five projection images each from three EM models that are not present in the 2D projection image library to test the match retrieval protocol (Fig. 7). EMD-3347 (proteasome) and EMD-2275 (80S ribosome) have highly similar models present in the expanded dataset, while the third, EMD-2326 (GroEL/ES chaperone complex), has no good 3D model match in the expanded dataset.

**Fig. 8:**
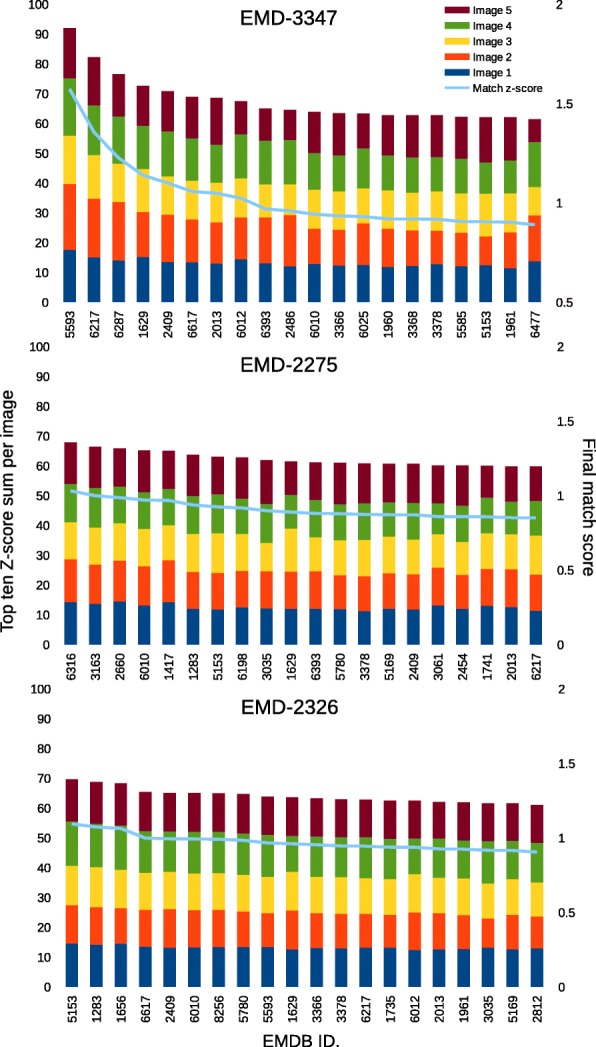
The top 20 model matches for EMD-3347, EMD-2275 and EMD-2326 with contributions to the score from each of the five input images. The stacked bar plot shows the top ten Z-score sum (*S*_*n*_ score) by input image (1 – blue, 2 – orange, 3 – yellow, 4 – green, 5 – maroon; left y-axis) for each of the top 20 model matches that are ordered by the final match score (*T*_*n*_ score; blue line; right y-axis)

In Fig. 8, the final match score *T*_*n*_ (Eq. 6) is represented as a blue line, and determines the ranking of the model matches. Here, we also see the values of *S*_*n*_ (Eq. 5) per image index, and find that in some cases, the individual input images have higher top 10 *S*_*n*_ scores than others. Such a scheme is useful for searching models that match a particular input image well, based on the 2D shape it contains. For example, in EMD-2326, the top scoring matches have larger contributions from input images 1 and 4, which are both ellipsoidal shapes whereas matches that have larger contributions from input images 2 and 3, such as EMD-1629 and EMD-6012, bear some similarity to the cylinder-like shape captured in those images (Fig. 7).

**Fig. 9:**
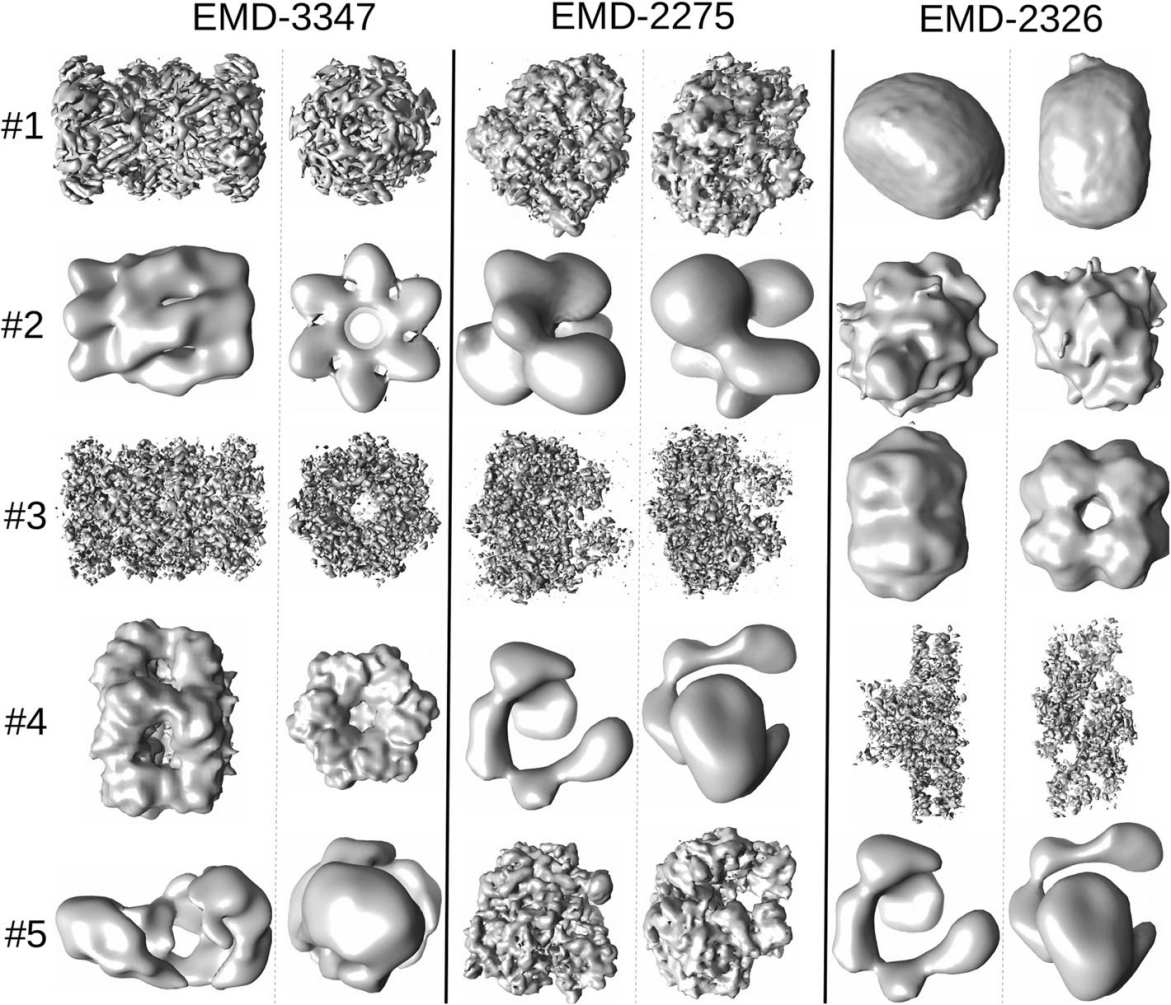
Top five search hits for each input model (EMD-3347, EMD-2275, EMD-2326). From rank #1 to # 5; EMD-3347: EMD-5593, EMD-6217, EMD-6287, EMD-1629, EMD-2409: EMD-2275: EMD-6316, EMD-3163, EMD-2660, EMD-6010, EMD-1417; and EMD-2326: EMD-5153, EMD-1283, EMD-1656, EMD-6617, EMD-6010

**Fig. 10:**
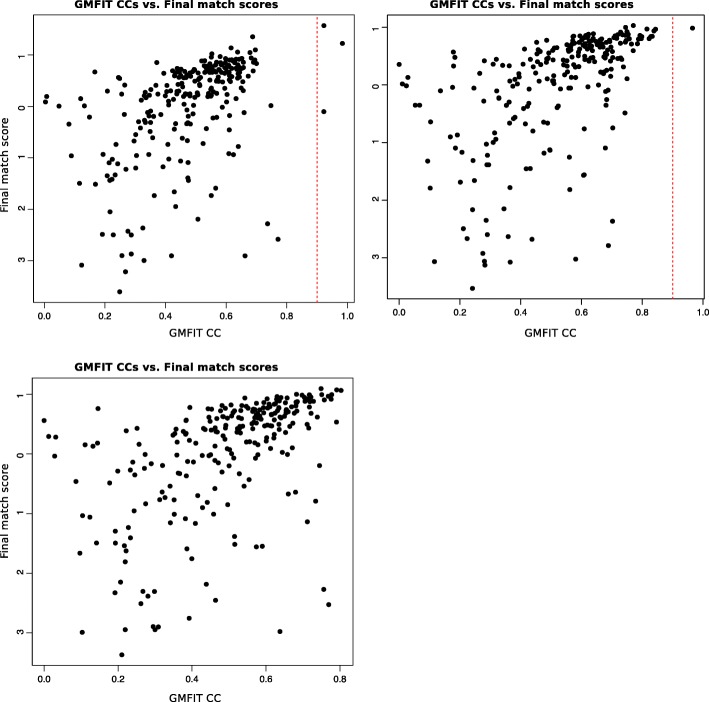
3D GMFIT correlation coefficients vs. final match scores (*T*_*n*_). In the case of EMD-3347 and EMD-2275, which have high similarity (GMFIT CC > 0.9, dotted red line) to at least one model in the projection image library, the corresponding final match score as retrieved using 5 input images in the search against the projection library is within the top 10 ranked hits. In EMD-2326, there is no highly similar model present

In the case of EMD-3347 and EMD-2275, we were able to retrieve the most similar 3D models within the first five hits for each (Fig. 9). For EMD-2326, no true match exists in the database. When we analyze individual images, we find that the top-ranking hit captures the cylindrical nature of the molecule, while the third ranking match resembles the lower half corresponding to the GroEL subunit of the original model. When we included the 2D projection image set generated from EMD-2326 in the projection library, and found that we were able to retrieve it as the top-ranking match using the same 5 input images as our test case, demonstrating that the inability to retrieve an accurate hit is not due to the design of the algorithm (Additional file 1: Figure S5). We find that the final match score, as calculated by using Eq. 6, is accurate when the GMFIT CCs between the 3D models of the test cases used and the 3D models in the expanded dataset are above 0.9 and they tend to correspond to top three match scores retrieved (Fig. 10). The top three ranking search matches for EMD-3347 have final match scores significantly higher than the rest, suggesting that a significant difference between two consecutive scores could be used to determine well-suited matches to the input data. However, as we have observed in Fig. 4, there are a large number of shapes that overlap with each other, largely corresponding to the ribosomal structures, resulting in a larger number of suitable 3D models being proposed with lesser difference between the final match scores. Finally, in the case of EMD-2326, even though some of the proposed 3D models capture features of the input images, due to the lack of a significantly well-matched model represented in the projection library, the final match scores are unable to indicate search matches that are more accurate than the rest. This requires a potential user to examine several top-ranking 3D shapes in the results to see if they possess common attributes, in order to assess their relevance to the data being analyzed. In general, the match retrieval protocol reveals that the success of the strategy defined here relies on the coverage of shapes within the projection image library.

## Discussion

In order to extract the overall 3D biological shape, we resized the EM models so that they have the same particle volume. We do this in order to decrease redundancy, and to normalize discrepancies between the models. By normalizing the volume in the database, we allow for the possibility that the shapes from diverse samples could be listed as potential shapes for the query images. This is especially useful for samples where a homologous structure may be unavailable, and yet their 2D images resemble shape of another molecule. This phenomenon is exemplified by the high similarity between the GMMs of the spherical Brome mosaic viral capsid (EMD-6000) and horse spleen apoferritin (EMD-2788) when their volumes are normalized.

Another purpose for removing redundant 3D biological shapes is to increase the efficiency of the search algorithm. Despite reducing the search space, we cannot avoid the large overlap we observe between many of the 2D projection images, which makes it difficult to distinguish between 3D models based on a single 2D image. In cases where the 3D shape is asymmetric, we observed greater heterogeneity in their corresponding 2D projection image sets. However, the individual 2D images have the potential to match many different 3D shapes. This led us to conclude that we would require a combination of 2D projection images to increase the possibility of capturing the overall 2D image profile belonging to particular 3D shape. The final match scores (Eq. 6) are normalized such that the contribution of each input image in the search are equalized (Eq. 5), and thus do not reflect on the exact quality of the match. This allows us to retrieve reasonable matches to our test examples and avoid the biasing effect of the highly overlapping 2D images to certain shape type, as observed in Fig. 5. However, depending on the case, the difference between the final match scores in the top ranking matches could indicate the quality of the match to the input, just as we observe in the results for EMD-3347 (Fig. 8). In general, that the lack of such final match score separation, as observed for EMD-2275 and EMD-2326, does not necessarily indicate low quality matches, requiring future users to compare several of the top ranking 3D models to the input data visually in order to assess their accuracy.

When we performed the test search for the three example targets, EMD-3347 (proteasome), EMD-2275 (80S ribosome) and EMD-2326 (GroEL/ES chaperone complex), the quality of the retrieved matches depended on the availability of highly similar structural alternatives in the database. Yet, in the case of EMD-2326, where no highly similar structure was present, we were able to identify shapes that corresponded to the outlines of each of the five input images; images 1, 4 and 5 contribute more to the top 3 ranking hits which have ellipsoidal and cylindrical shapes while images 2 and 3 contribute less due to the absence of similar “bullet-shaped” models in the projection library. In summary, our results indicate that with sufficient coverage in shape types in the projection library, we will be able to provide an idea of the 3D shape captured by the input image more reliably.

We find that this hybrid approach allows for many potential applications. Firstly, we envision that some EM or XFEL data that might not be good enough for 3D reconstruction still contains useful information about the 3D structure of the sample of interest, and thus obtaining a possible idea about the 3D shape could be a useful start. In some cases, producing a 3D structure with atomic-level resolution is not the only use for EM as an experimental technique. For example, 2D negative stain EM images have been used to gain insight into the functional complex formation of the mammalian circadian clock proteins in the cell [42]. Our aim is to provide such an alternative tool to obtain new information from the experimental data.

In the future, we aim to expand our projection library to include 3D shapes gathered from the Protein Databank (PDB) [43], SASDB [44] and the rest of the Electron Microscopy Databank (EMDB) [38]. This approach is analogous to other webservers such as DARA [34] and SASTBX [35] for searching initial SAXS models, EM-surfer [45] for comparing a particular EM model to a database of EM surfaces, and Omokage search [39], for searching 3D structures across all the different structure databases and retrieving different similar shape types across different functions and resolutions. When performing large-scale structural data analysis, our strategy can be part of an integrative structural biology approach [46]. The visual proteomics approach promises to create a “molecular atlas” of the cell by fitting individual template structures into an electron tomography map of cell [47, 48]. In visual proteomics, 3D template matching is used to assign structures to the electron densities in a tomogram [49, 50]. Our strategy could be used to identify cellular components, in various orientations, in 2D electron microscopy data. It could also be used to quickly estimate the mixing of states or conformations that can be present within the experimental data [51].

For the performance of the match retrieval, a stack of 7544 images takes approximately 7.2 min using a single core of Intel Xeon E5–2650 V2 to align against a single input reference image. Using a cluster with multiple nodes and by dividing the image library into three stacks that can be aligned against the input query images as references concurrently, a search with one input image can be performed in approximately 1 h. We plan to increase the speed of the calculations by reducing the number of 2D images simulated per model in the library, and to pre-compute the similarity between the images so that quick neighbor searches can be performed.

## Conclusion

In this study, we were able to assemble a collection of 3D biological shapes by resizing known EM structures to the same relative size, and then comparing them by treating them as GMMs. Furthermore, when we simulated 2D images from the 3D shapes, we found that there can be a large overlap between many of the images from different models. Yet, depending on the complexity of the given model’s shape, the corresponding 2D projection images can be highly heterogeneous. Still, the algorithm we have proposed in this study is able to determine the 3D shape that matches a low number (5) of query images, when searching against a library of 2D projection images from 250 EM models. Our strategy can find potential 3D shapes from experimental data without considering volume information. In the future, we will expand the number of biological shapes represented in the 2D image library using data from other structure databases like the PDB, EMDB and SASBDB, and make it available as a webserver for the structural biology community. Lastly, the applications of our hybrid approach are numerous, whether it is used to quickly identify possible shapes for novel single particle data, to estimating the number of conformations that can be present in experimental data from EM or XFEL.

## Additional file

Additional file 1: **Table S1.** List of EMDB IDs in both small (25 models) and expanded dataset (250 models). **Figure S1.** Distribution of single particle EMDB entries according to number of components A), resolution B) and structure type C). **Figure S2.** Multidimensional scaling plot zoomed in to present the top 3 most populated cells with the following axis boundaries: cell 33 (− 0.2 < = x < − 0.1, 0 < = y < 0.1), cell 59 (0.1 < = x < 0.2, − 0.1 < =y < 0) and cell 60 (0.1 < −x < 0,2, 0 < =y < 0.1). The cells are split further into 0.05 (x axis) by 0.02 (y axis) subcells. Each representative image has the highest occurring EMDB ID in each subcell. **Figure S3.** Structure types in A) All Single Particle data in the EMDB, (B) Reduced Single Particle data based on 3D analysis and C) in the randomly expanded EMDB data. **Figure S4.** Gaussian kernel density plots illustrating the distribution of 2D image alignment correlation coefficients (CCs) from the small dataset, with 406, 203, 101 and 58 different 2D projection images per EM model. For each number of 2D projection images used, we first calculated the submatrix of CCs between images for one EM model against images from all EM models (for example, 406 by 10150 (=406 × 25) CCs). Then we calculated the kernel densities of the submatrices associated with each EMDB ID. The plots show that there is no change in the position of the peaks, which means that the distribution of the scores remains consistent. **Figure S5.** The top 50 model matches for EMD-2326, when performing the search using 5 input images against the 2D projection image library generated from the expanded dataset of 250 EM models and EMD-2326 (251 models in total). (PDF 2600 kb)

## Abbreviations

2D: Two-dimensional
3D: Three-dimensional
CC: Correlation coefficient
EM: Electron Microscopy
EMDB: Electron Microscopy Database
EMPIAR: Electron Microscopy Pilot Image Archive
GDF: Gaussian distribution function
GMM: Gaussian mixture model
MDS: Multidimensional scaling
PCC: Pearson’s correlation coefficient
PDB: Protein Databank
SASDB: Small Angle Scattering Biological Databank
SAXS: Small angle x-ray scattering
XFEL: X-ray free electron laser
