# Supplementary material for: Searching for 3D structural models from a library of biological shapes using a few 2D experimental images
Source: BMC Bioinformatics. 2018 Sep 12;19:320. doi: 10.1186/s12859-018-2358-0 (PMC6134691; doi:10.1186/s12859-018-2358-0)
Supplement: Supplementary file 1 — Table S1. List of EMDB IDs in both small (25 models) and expanded dataset (250 models). Figure S1. Distribution of single particle EMDB entries according to number of components A), resolution B) and structure type C). Figure S2. Multidimensional scaling plot zoomed in to present the top 3 most populated cells with the following axis boundaries: cell 33 (− 0.2 < = x < − 0.1, 0 < = y < 0.1), cell 59 (0.1 < = x < 0.2, − 0.1 < =y < 0) and cell 60 (0.1 < −x < 0,2, 0 < =y < 0.1). The cells are split further into 0.05 (x axis) by 0.02 (y axis) subcells. Each representative image has the highest occurring EMDB ID in each subcell. Figure S3. Structure types in A) All Single Particle data in the EMDB, (B) Reduced Single Particle data based on 3D analysis and C) in the randomly expanded EMDB data. Figure S4. Gaussian kernel density plots illustrating the distribution of 2D image alignment correlation coefficients (CCs) from the small dataset, with 406, 203, 101 and 58 different 2D projection images per EM model. For each number of 2D projection images used, we first calculated the submatrix of CCs between images for one EM model against images from all EM models (for example, 406 by 10150 (=406 × 25) CCs). Then we calculated the kernel densities of the submatrices associated with each EMDB ID. The plots show that there is no change in the position of the peaks, which means that the distribution of the scores remains consistent. Figure S5. The top 50 model matches for EMD-2326, when performing the search using 5 input images against the 2D projection image library generated from the expanded dataset of 250 EM models and EMD-2326 (251 models in total). (PDF 2600 kb) [file 12859_2018_2358_MOESM1_ESM.pdf]

Supplementary Material

Searching for 3D structural models from a library of biological shapes using a few 2D experimental images

Sandhya P. Tiwari[1], Florence Tama[1,2*], Osamu Miyashita[1]

[1]Computational Structural Biology Unit, RIKEN Advanced Institute for Computational Science, Japan

[2] Graduate School of Science, Department of Physics & Institute of Transformative Bio-Molecules (WPI-ITbM), Nagoya University, Japan

*** Correspondence:** florence.tama@riken.jp

Supplementary Table 1 - List of EMDB IDs in both small (25 models) and expanded dataset (250 models)

Models from small dataset	Models from rest of EMDB		
3233	**6340**	**1102**	**6014**
3187	**2295**	**5806**	**2812**
2981	**1021**	**5921**	**2048**
6392	**8101**	**8234**	**5293**
6393	**1172**	**3201**	**8118**
3035	**1857**	**3177**	**3378**
2660	**1325**	**6217**	**1393**
6287	**5131**	**1192**	**1410**
2852	**2975**	**3078**	**1107**
5447	**5118**	**1839**	**2466**
5778	**2319**	**1199**	**6416**
2484	**2015**	**2077**	**5505**
	**6012**	**6575**	**1706**
	**1413**	**3366**	**1123**

	**5231**	**1727**	**2658**
	**2454**	**1961**	**5436**
	**6316**	**2697**	**1018**
	**5195**	**5822**	**1424**
	**2871**	**5134**	**2553**
	**1656**	**5604**	**5169**
	**8103**	**6589**	**1048**
	**3207**	**3061**	**5173**
	**6432**	**5670**	**1928**
	**6321**	**3376**	**5809**
	**2354**	**5618**	**5182**
	**1218**	**2492**	**1741**
	**6555**	**1175**	**1526**
	**1894**	**3295**	**5431**
	**1187**	**8001**	**1874**
	**6056**	**5600**	**6132**
	**2030**	**8256**	**5937**
	**1230**	**6369**	**1283**
	**6025**	**5500**	**3034**
	**1962**	**2031**	**2539**
	**2311**	**3186**	**2195**
	**2013**	**5381**	**2206**
	**2053**	**1804**	**2817**
	**5960**	**6581**	**2688**
	**5443**	**5671**	**5113**
	**1153**	**6421**	**1054**
	**1005**	**1023**	**6382**

	1417	2310	8140
	8167	5593	1912
	3282	5501	6385
	4002	9507	6224
	5680	6010	3368
	1776	1761	2537
	1821	5116	3324
	2685	5143	6035
	1167	6086	1942
	2786	6617	6409
	5720	5996	6209
	6418	1109	1672
	5385	1190	2067
	2478	6150	5434
	2224	1256	1188
	6057	5981	2409
	2170	5108	1629
	1765	6198	3073
	6558	5445	1403
	6477	5392	5251
	1603	6488	3301
	1881	2435	2207
	8208	8235	5430
	1640	1921	2540
	3143	1729	1523
	2486	5585	1960
	5916	6329	5140
	1612	1725	2282

	**2464**<br>**5580**<br>**2208**<br>**1213**<br>**1735**<br>**2820**<br>**2536**<br>**1481**<br>**2283**<br>**1042**<br>**5780**	**2808**<br>**5584**<br>**2481**<br>**5408**<br>**3077**<br>**2990**<br>**5034**<br>**8164**<br>**8205**<br>**1138**	**3163**<br>**1157**<br>**8168**<br>**5153**<br>**6301**<br>**6440**<br>**5864**<br>**1617**<br>**1670**<br>**1333**

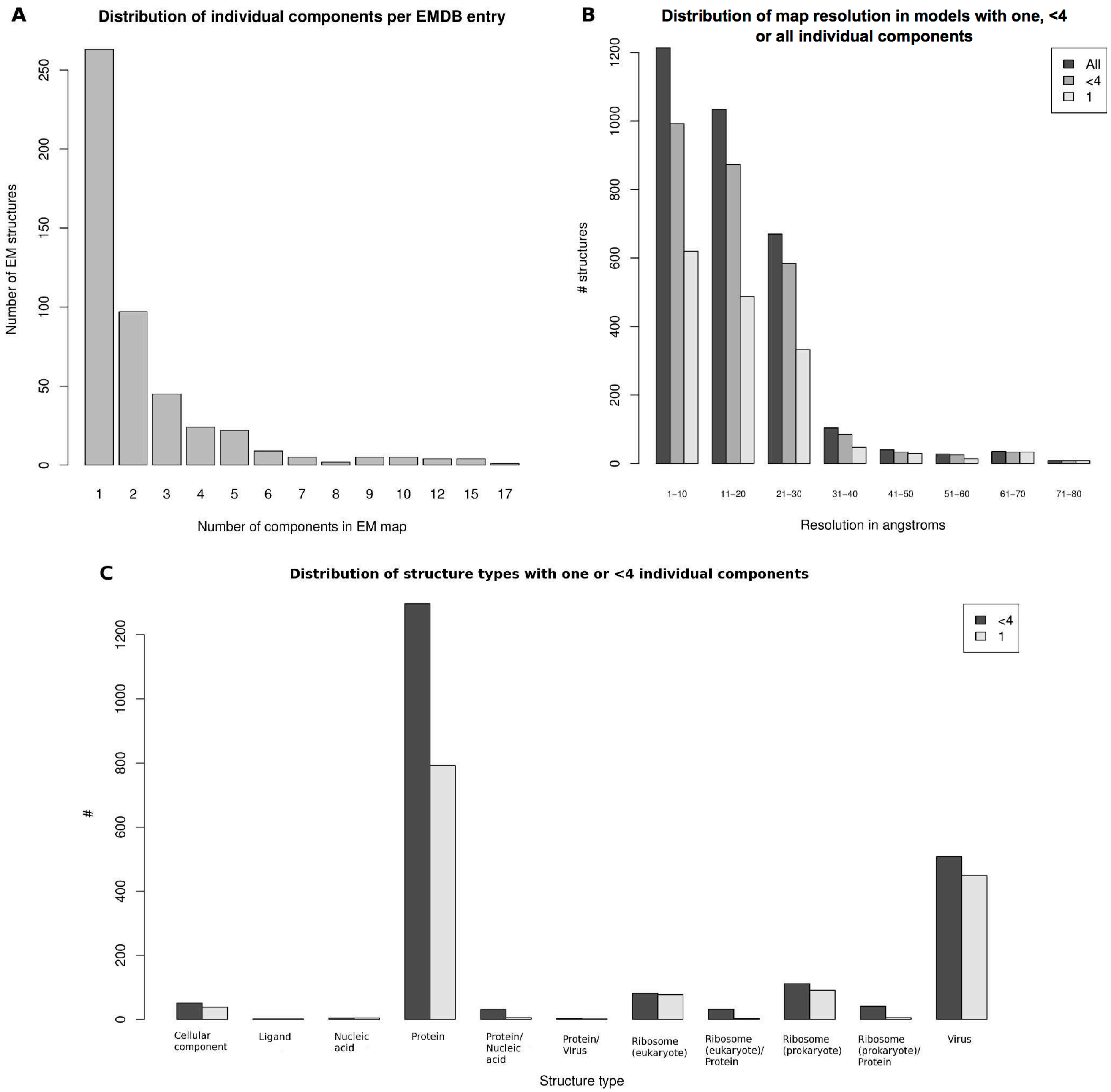

Supplementary Figure 1 - Distribution of single particle EMDB entries according to number of components A), resolution B) and structure type C).

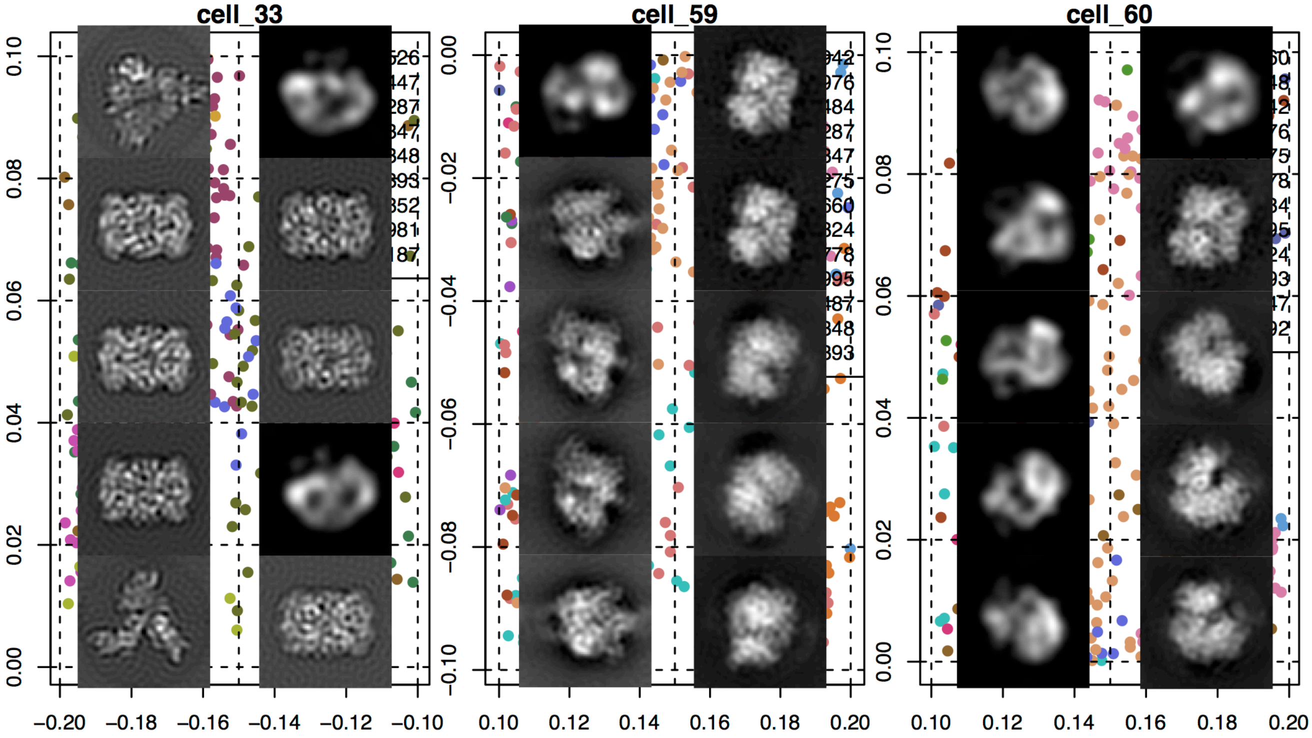

Supplementary Figure 2 - Multidimensional scaling plot zoomed in to present the top 3 most populated cells with the following axis boundaries: cell 33 (-0.2 <= x < -0.1, 0<= y < 0.1), cell 59 (0.1 <= x < 0.2, -0.1 <=y<0) and cell 60 (0.1 <-x<0,2, 0<=y<0.1). The cells are split further into 0.05 (x axis) by 0.02 (y axis) subcells. Each representative image has the highest occurring EMDB ID in each subcell.

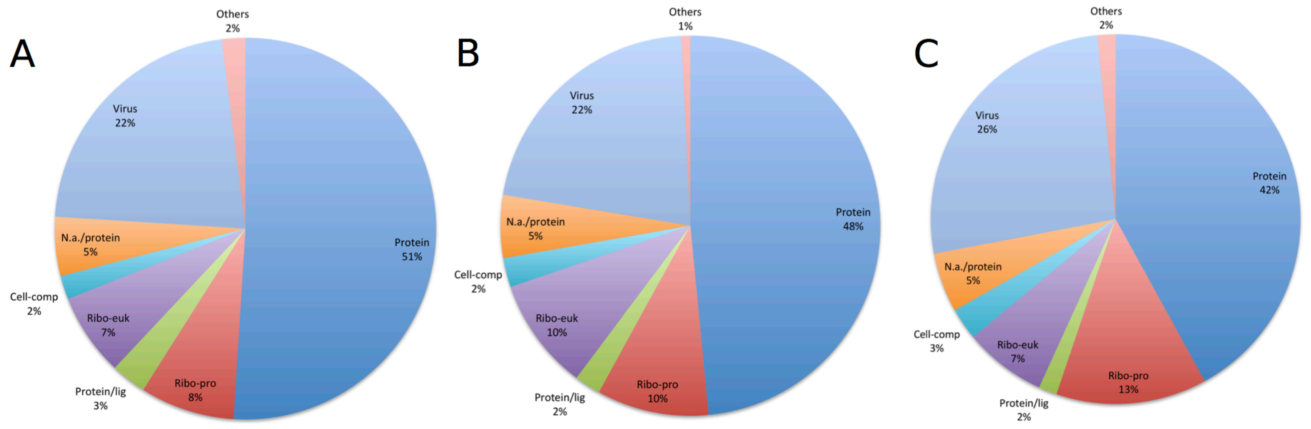

Supplementary Figure 3 - Structure types in A) All Single Particle data in the EMDB, (B) Reduced Single Particle data based on 3D analysis and C) in the randomly expanded EMDB data.

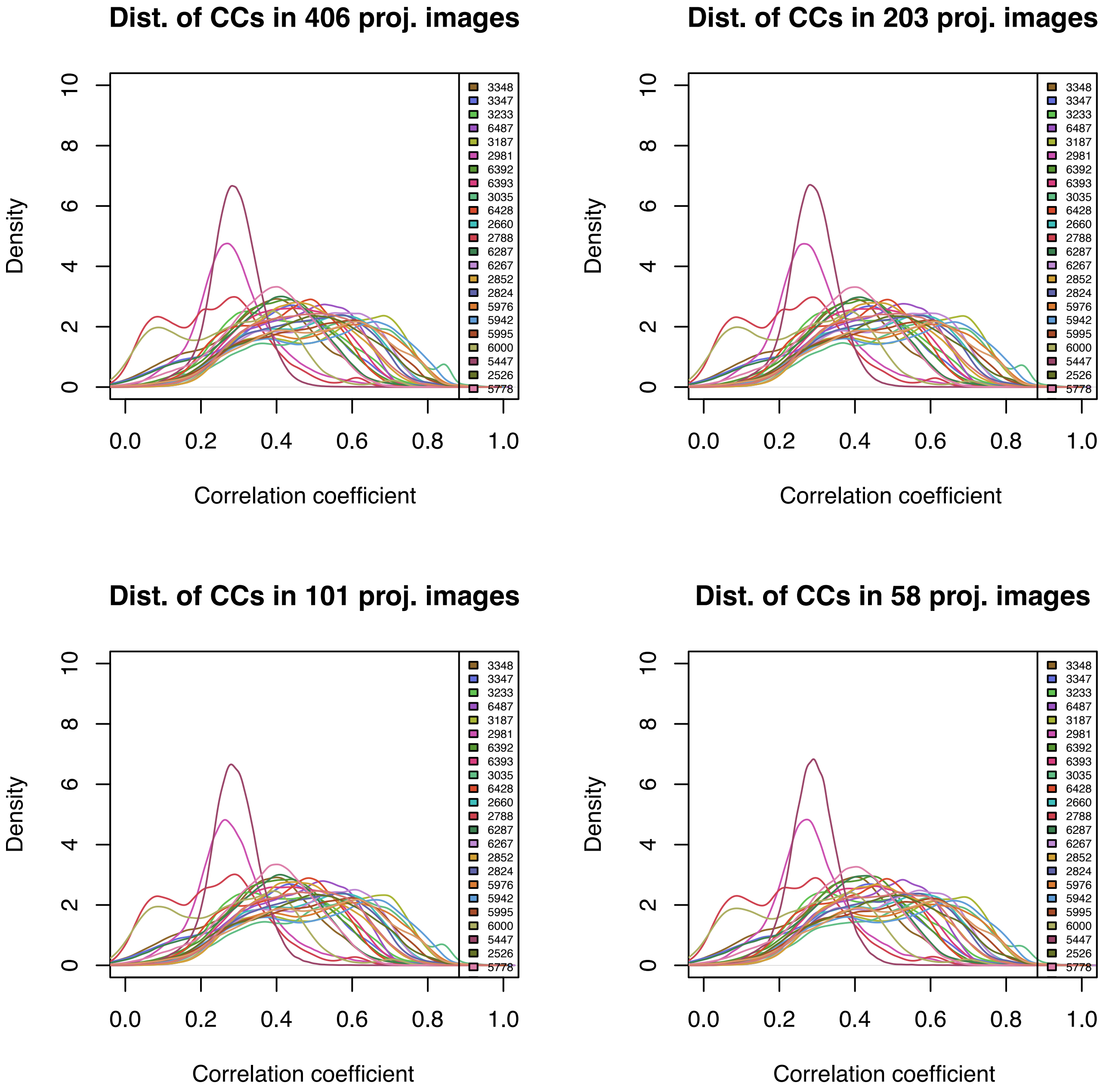

Supplementary Figure 4 – Gaussian kernel density plots illustrating the distribution of 2D image alignment correlation coefficients (CCs) from the small dataset, with 406, 203, 101 and 58 different 2D projection images per EM model. For each number of 2D projection images used, we first calculated the submatrix of CCs between images for one EM model against images from all EM models (for example, 406 by 10150 (=406x25) CCs). Then we calculated the kernel densities of the submatrices associated with each EMDB ID. The plots show that there is no change in the position of the peaks, which means that the distribution of the scores remains consistent.

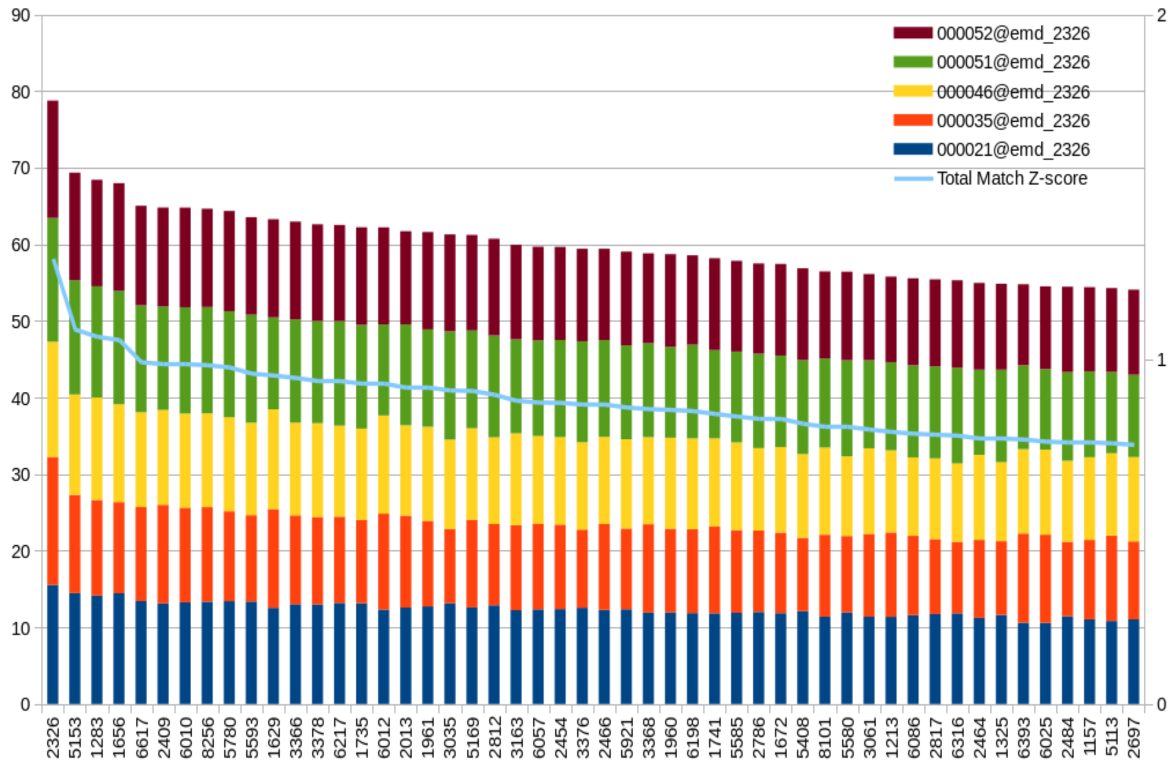

Supplementary Figure 5 – The top 50 model matches for EMD-2326, when performing the search using 5 input images against the 2D projection image library generated from the expanded dataset of 250 EM models and EMD-2326 (251 models in total). The stacked bar plot shows the top ten Z-score sum (S_n score) by input image (1 – blue, 2 – orange, 3 – yellow, 4 – green, 5 – maroon; left y-axis) for each of the top 20 model matches that are ordered by the final match score (T_n score; blue line; right y-axis).
